# Barriers and enablers to cognitive assessment in Parkinson's disease: A qualitative contextual inquiry

**DOI:** 10.1177/1877718X261440707

**Published:** 2026-04-27

**Authors:** Deborah Brooks, Kirstine Shrubsole, Deepa Sriram, Jihyun Yang, Leander K. Mitchell, Peter Worthy, Elton Lobo, Dana Pourzinal, Nadeeka N. Dissanayaka

**Affiliations:** 1 UQ Centre for Clinical Research, The University of Queensland, Herston, Queensland, Australia; 2 Queensland Aphasia Research Centre, School of Health and Rehabilitation Sciences, The University of Queensland, Herston, Queensland, Australia; 3 School of Psychology, The University of Queensland, St Lucia, Queensland, Australia; 4 School of Electrical Engineering and Computer Science, The University of Queensland, St Lucia, Queensland, Australia

**Keywords:** cognitive assessment, Parkinson's disease, mild cognitive impairment, dementia, qualitative inquiry

## Abstract

**Background:**

Cognitive impairment in Parkinson's disease (PD) is often overlooked, despite increased risk of dementia in PD. ‘PDCogniCare’ is an innovation aiming to improve access to cognitive assessments, for earlier diagnosis and care. This study explored barriers and enablers to cognitive assessment in PD, to inform implementation of ‘PDCogniCare’.

**Methods:**

Participants included ten people with PD, one caregiver, and nineteen health professionals within two Australian public health services. Semi-structured interviews were informed by the Theoretical Domains Framework (TDF) and the Consolidated Framework for Implementation Research (CFIR). Deductive and inductive analysis was utilised. Barriers were mapped to the CFIR-ERIC (Expert Recommendations for Implementing Change) matching tool to develop implementation strategies.

**Results:**

Seven themes were identified: 1) lack of discussion about cognitive impairment limits access to cognitive assessments and interventions; 2) clinicians need to ‘be on board with cognition’; 3) availability of clinic resources impacts delivery of cognitive screens and assessments; 4) variability of clinician decision-making processes to screen or refer patients for neuropsychological assessment; 5) impact of undergoing neuropsychological assessments on people living with PD; 6) uncertainty over benefits of cognitive assessment in changing clinical management; and 7) perceived advantages of ‘PDCogniCare’ over current practice. Strategies to address identified barriers included: clinician education and training, additional resource allocation, support and feedback, and involving patient/consumer advocates.

**Conclusions:**

Several barriers and enablers to cognitive assessment in PD were identified, relating to health professional knowledge, beliefs, context and resources. Strategies that address these may improve clinical practice for more proactive treatment and care.

## Introduction

Parkinson's disease (PD) is the second most common neurogenerative disorder after Alzheimer's disease, with prevalence estimates increasing to 15 million people by 2030.^1^ Whilst PD is characterised by its motor symptoms, cognitive impairment and dementia are common non-motor features; at least 75% of people living with PD will develop dementia after 10 years.^2^ Mild cognitive impairment in PD (PD-MCI) is associated with a greater risk of developing dementia, and present in 20–30% of people upon diagnosis of PD.^2^ PD-MCI and PD-dementia negatively impact quality of life, reduce function, increase caregiver strain and increase costs to the person, their caregivers and healthcare systems.^3–6^

Globally, national dementia action plans recommend timely diagnosis of dementia to ensure people receive appropriate treatment, care and support.^7^ Numerous benefits of early diagnosis include involvement in decision-making, legal, financial and care planning, accessing pharmacological and non-pharmacological treatments and trials, formal and informal support networks and improved quality of life.^8^ A UK survey reported that over 90% of people impacted by dementia felt that early diagnosis was beneficial, and 60% wished they had received a diagnosis earlier.^9^ Cognitive assessments are recommended as part of the diagnostic pathway.^10^ Commonly used brief cognitive assessments/screens include the Mini Mental State Examination (MMSE)^11^ and the Montreal Cognitive Assessment (MoCA).^12^ In-depth neuropsychological assessment may be indicated for diagnostic accuracy in complex cases and can provide additional benefits regarding the management of cognitive, functional and neuropsychiatric symptoms.^13^ The routine delivery of brief and comprehensive cognitive assessments can enable early identification of cognitive and functional decline, monitor progression over time and facilitate proactive clinical and personal decision-making regarding treatment, care and support.^14^ However, despite the prevalence of MCI at PD diagnosis and high risk of developing dementia, best practice guidance for neuropsychological assessment is limited in this population.^15^

Due to the initial predominance of motor features (e.g., tremors, muscle rigidity), people living with PD are usually treated under the care of neurologists or movement disorder specialists.^3,16^ Awareness of cognitive impairment in these settings is often poor,^3^ even though people living with PD report maintenance of cognitive function as a major unmet need.^4^ This may be because the diagnosis of PD-MCI and PD-dementia remains challenging in practice due to difficulty characterising functional impairment, variability in neuropsychological test selection, and heterogeneity of cognitive phenotypes.^17^ As PD is a complex condition requiring multidisciplinary care, lack of connection between services (primary and secondary, as well as neurology, allied health and mental health services) has meant that patients and their families are “falling through the gaps”.^3^

To address these issues, our ‘PDCogniCare’ study,^18^ has developed best practice guidelines for diagnosis, cognitive evaluation, and management of cognitive disorders in PD,^14,19^ inclusive of an evidence-based neuropsychological toolkit specific to PD.^20^ The co-design and development of a digital ‘PDCogniCare’ platform offers opportunities to deliver training on the best practice guidelines for health professionals working within neurology or movement disorders clinics, and the neuropsychological toolkit for psychology services. It will also manage referrals between services, track cognitive assessment results over time and facilitate sharing of results between health professionals, patients and family caregivers.^21^

The pre-implementation study reported here, aims to understand factors likely to influence implementation of ‘PDCogniCare’ within partnered neurology, movement disorders, psychiatry and psychology clinics across two metropolitan health services in Australia. These will inform the development and subsequent testing of implementation strategies. A thorough understanding of contexts and identification of barriers and enablers is vital for an innovation's successful and sustainable implementation.^22^ Specific research questions were:

- What are the barriers and enablers to delivering or receiving a brief cognitive screen/comprehensive cognitive assessment from the perspectives of people living with PD and clinical stakeholders?
- What are the potential barriers and enablers to implementing the proposed ‘PDCogniCare’ platform within current services from the perspectives of people living with PD and clinical stakeholders?
- What implementation strategies can be used to address the identified barriers and leverage enablers?

## Methods

### Research design

This was a pre-implementation qualitative contextual inquiry study, utilising interviews to collect data.^23^ The study was approved by Metro North Human Research Ethics Committee (HREC/2023/MNHA/100098) and The University of Queensland Human Research Ethics Committee (2023/HE002029). The Consolidated Criteria for Reporting Qualitative Research (COREQ) were used to ensure comprehensive reporting and transparency (see Additional file 1).^24^

### Sample and recruitment

Purposive sampling strategies were used, aiming for maximum variation in roles as recommended for implementation qualitative research.^25^ The sample included any stakeholders involved in screening or assessing for cognitive impairment in PD public hospital clinics (e.g., neurology, movement disorders, psychology and psychiatry clinics). Eligible participants included people living with PD, and neurologists, medical registrars, psychiatrists, geriatricians, neuropsychologists, movement disorders nurses, and allied health professionals from two health services in metropolitan Queensland, Australia. We aimed to recruit 10 health professionals from each health service setting and 10 people living with PD (total N = 30), as per recommendations for an implementation-based qualitative interview study.^25^ Participants needed to be aged over 18 years and able to communicate in English to take part. Participants were offered a $50 e-gift voucher in recognition of their time.

Stakeholder mapping activities identified potential health service participants to be invited for interview.^26^ These participants were asked to identify other key stakeholders to ensure that no relevant individuals from their health service or clinic had been missed (i.e., snowball sampling). People living with PD were recruited via adverts and talks in the community, and our existing PD research participant database. Recruitment continued until a sufficient range of roles and experiences were included, and no new themes were identified from the data. All participants received an information sheet describing the study and were given the opportunity to ask questions before signing a consent form. Verbal consent was re-established at the beginning of each interview.

### Theoretical frameworks

We developed two versions of an interview guide (see Additional files 2 and 3). The interview guide for people living with PD was informed by the Theoretical Domains Framework (TDF) of behaviour change to inform implementation.^27^ The interview guide for clinicians and health professionals was informed by both the TDF and the Consolidated Framework for Implementation Research (CFIR) version 1.^28^ The TDF primarily considers *individual determinants* and includes 14 constructs relating to knowledge, skills, social/professional role and identity, beliefs about capabilities, optimism, beliefs about consequences, reinforcement, ​intentions​, goals, decision processes, environmental context and resources, social influences​, emotion​ and behavioural regulation​. The CFIR primarily considers *organisational determinants* used across many healthcare settings and provides a list of defined potential implementation determinants across five domains: (i) intervention to be implemented; (ii) inner and (iii) outer setting; (iv) individuals involved; and (v) implementation process. Both implementation frameworks are well-operationalized, and whilst there is overlap in some constructs, they can be used in combination to more fully understand behaviour change within organisations and develop implementation strategies tailored to context.^29^

### Data collection

Semi-structured interviews were conducted via video-conferencing platforms (e.g., Zoom or TEAMS), 45–60 min in duration, and audio-recorded with consent for professional transcription purposes. Interviews were conducted by individual members of the research team (DB, PW, KS, JY, LM). Seven of the clinician/health professional participants were known to the interview team prior to participation. Whilst a prior relationship can help build rapport, all interviews followed the semi-structured interview guide (Additional files 2 and 3), as a ‘frame of activity’ and means of mitigating any bias that a prior relationship may have incurred.^30^ To ensure consistency, the first interview recording was watched and discussed by interviewers in a subsequent meeting. Interview guide topics included: (i) exploration of current clinical practice and patient experience with regards to cognitive assessment and use of assessment results within clinics; (ii) potential advantage of the proposed system over current practice and fit into existing systems; (iii) individual motivation, values, and beliefs; (iv) existing skills, knowledge, time and resources; (v) learning environment; (vi) influence of colleagues and local opinion leaders; and (vii) leadership support, power and authority. Both interview guides included showing a short video explaining the proposed ‘PDCogniCare’ platform for improving access to cognitive assessments in PD. Findings relating to technology issues have been reported elsewhere.^21^

### Data analysis

Data analysis primarily utilised a deductive framework analytic approach informed by the CFIR and TDF domains and constructs. Inductive analysis was also conducted to identify codes not mapped within the CFIR and TDF frameworks.^31^ Transcripts were uploaded into NVIVO analysis software for coding. Two researchers (DS, DB), independently coded the transcripts. Coding included consideration of participants’ responses within each transcript, and its relevance to one or more pre-defined CFIR domains or TDF constructs (using codebooks with definitions of each). Where data did not fall within the predefined frameworks, emerging codes were added. Codes were further categorized into barriers or enablers and used to construct themes. A meeting to discuss and resolve any coding discrepancies was also held (DS, DB, KS). We then used the CFIR-ERIC (Expert Recommendations for Implementing Change) Matching Tool, to identify implementation strategies which may mitigate the identified barriers.^32,33^ Members of the research team (DB, KS, DS, EL, DP) met to discuss and agree upon the key strategies for the implementation of ‘PDCogniCare’ in a future trial.

## Findings

### Participants

A total of 30 participants took part in the interviews. These included 19 clinicians and health professionals recruited from the two metropolitan health services in Australia (Site A n = 9, Site B n = 10). We also recruited 10 people living with PD (and one caregiver as a joint interview). The characteristics of participants are shown in Tables 1 and 2.

**Table 1.** Clinical and health professional participant characteristics (N = 19).

Variable	N	%
**Professional Role**		
Movement Disorders Clinic Nurse	2	10.5
Neurologist	4	21.0
Psychiatrist	2	10.5
Geriatrician	1	5.2
Neuropsychologist	5	26.3
Speech Pathologist	3	15.8
Occupational Therapist	1	5.2
Physiotherapist	1	5.2
**Years in current role**		
1–4	5	26.3
5–9	6	31.6
10–14	1	5.2
15–19	1	5.2
20–24	3	15.8
25–29	1	5.2
Not reported	2	10.5
**Gender**		
Female	11	58
Male	8	42
**Age**	**Mean**	**Range**
	47	33–68

**Table 2.** Lived experience participant characteristics (N = 11).

Variable	N	%
**Lived Experience Expert**		
Person with PD	10	91
Family caregiver	1	9
**Gender**		
Female	5	45
Male	6	55
**Age**	**Mean**	**Range**
	67	50–77

### Themes

The following seven themes were constructed from the qualitative data. Table 3 shows how the themes mapped to the TDF, CFIR and ERIC strategies, as well as the source of the theme.

**Table 3.** Themes mapped to the TDF, CFIR and ERIC implementation strategies.

Theme	Perspectives	TDF constructs	CFIR domains	ERIC strategies
Lack of discussion about cognitive impairment limits access to cognitive assessments and interventions	- People living with PD - Neuropsychologists	- Knowledge - Skills - Beliefs about consequences	- Individual characteristics: - Knowledge and beliefs	- Develop educational materials - Prepare patients/consumers to be active participants - Obtain and use patients/consumers and family feedback
Clinicians need to be ‘on board with cognition’	- All	- Knowledge - Professional roles	- Individual characteristics: - Knowledge and beliefs	- Develop educational materials - Conduct educational meetings - Conduct ongoing training
The availability of resources (staff, time) impacts delivery of cognitive screens and comprehensive neuropsychological assessments	- Clinicians and health professionals	- Environmental context and resources - Professional roles	- Innovation: - Costs - Readiness for implementation: - Available resources	- Access new funding and allocation of resources
Clinician decision-making processes to screen or refer for neuropsychological assessment vary	- Clinicians and health professionals	- Knowledge - Skills - Decision processes	- Innovation: - Evidence strength and quality - Implementation climate: - Compatibility - Individual characteristics: - Knowledge and beliefs	- Develop educational materials - Conduct educational meetings - Conduct ongoing training
The impact of undergoing neuropsychological assessments on people living with PD	- All	- Emotion - Beliefs about consequences	- Innovation: - Relative advantage - Implementation climate: - Beliefs about the innovation	- Develop educational materials - Prepare patients/consumers to be active participants - Obtain and use patients/consumers and family feedback
Uncertainty over benefits of cognitive assessment in changing clinical management	- Clinicians and health professionals	- Beliefs about consequences - Professional roles	- Innovation: - Evidence strength and quality - Relative advantage - Implementation climate: - Tension for change - Beliefs about the innovation	- Develop educational materials - Conduct educational meetings - Identify and prepare champions - Prepare patients/consumers to be active participants - Obtain and use patients/consumers and family feedback
Perceived advantages of PDCogniCare over current practice.	- All	- Beliefs about consequences - Professional roles - Optimism	- Innovation: - Relative advantage - Complexity - Design - Readiness for implementation: - Available resources - Individual characteristics: - Personal attributes (motivation, capacity)	- Identify and prepare champions - Organize clinician implementation team meetings - Provide local technical assistance - Remind clinicians - Audit and provide feedback

**Note:** CFIR **=** Consolidated Framework for Implementation Research; ERIC **=** Expert Recommendations for Implementing Change; TDF = Theoretical Domains Framework

#### Lack of discussion about cognitive impairment limits access to cognitive assessments and interventions

Participants in both groups identified that, in neurology clinics, the main focus of PD information and assessment is on physical (rather than cognitive) symptoms and pharmacological (rather than non-pharmacological) treatments. Participants living with PD stated they had received little information about cognitive impairment from their clinicians. This put the onus onto the person with PD and their family to be able to find and understand relevant information themselves, often online or via PD support groups.

“*You’ll get this line which is a couple of words that talks about mild cognitive impairment. And at the time, I was like, well, I don’t really know what that means*.” (Person living with PD, ID01, quote represents a perceived barrier)

Inevitably, it was believed that lack of discussion about cognition with clinicians limited access to clinical and neuropsychological services. Some participants with PD noted they had only been able to access cognitive assessments via research projects or by paying for a private neuropsychological assessment (self-referral). Lack of referrals was a barrier to accessing psychological and cognitive interventions that may be beneficial for people living with PD.

“*I've specific examples where patients have chosen not to take medication. And so the neurologist says, okay, we'll see you in one year. Nothing else is done for this patient. And when in one year the patient goes and they still don't want medication they're told, well, I think I can discharge you then, there's nothing I can offer you. Which I think is severely wrong because, you know, there's so much we can offer people from a psychology perspective and neuropsychology perspective that is being completely neglected.”* (Neuropsychologist, Staff ID14, quote represents a perceived barrier)

#### Clinicians need to be ‘on board with cognition’

From the perspectives of people living with PD and some neuropsychologists, the lack of discussion about cognitive impairment and referrals for cognitive assessments stemmed from clinicians (e.g., neurologists, registrars or other medical specialists) not being engaged in cognitive symptoms.

“*It wasn’t that accessible. I saw two neurologists. I’ve stayed with one of them. They still, to this day, do not really engage particularly with the cognitive side of Parkinson's.”* (Person living with PD, ID01, quote represents a perceived barrier)

“*It's almost like that's not a focus for the neurologist, it's the movement symptoms that are the concerns. It's not their cognition that they're worried about. It's only when they were operating, they say, oh, let's look at the cognition. And then they will refer. So I feel like that's more the reason why we haven't been getting the referrals.”* (Neuropsychologist, Staff ID13, quote represents a perceived barrier)

However, many clinicians and health professionals reported mixed awareness of current guidelines or recommendations for the referral or delivery of cognitive assessments in PD. Conversely, some clinicians reported that they were actively monitoring the non-motor symptoms of PD, including cognitive issues, as the disease progressed.

“*If it's self-reported but also it's something I try to routinely ask for in my review patients as part of the questions for non-motor symptoms of which obviously cognitions are major one and the other things that might flag some underlying cognitive issues, if they've got hallucinations, developing or recent delirium and the setting of some medical issue. And I guess as a disease, particularly in the more advanced stages, I'm a bit more vigilant forward as well the cognitive issues*.” (Neurologist, Staff ID02, quote represents a perceived enabler)

#### Availability of clinic resources impacts delivery of cognitive screens and assessments

People living with PD reported they may only have a short appointment with a specialist clinician (such as a neurologist) once or twice a year, which was felt to be insufficient to discuss cognitive as well as physical changes and medications.

“*The last one I had, neurologist had a sign up that said, Dr X sees his patients for 20 min twice a year. Okay. No, I don’t want to see that sign. It was put up after I started seeing him, and no, I need more than that. I need more than 20 min twice a year.”* (Person living with PD, ID03, quote represents a perceived barrier)

Public health outpatient clinics were also running at full capacity, with time being a key barrier to the delivery of cognitive screens and assessments.

“*It depends on individual circumstances. The clinic is often over-booked, and they are short on time. Cognitive assessments are not routinely conducted for most patients.”* (Neurologist, Staff ID03, quote represents a perceived barrier)

However, there was variability between the two public health services clinics in terms of staff available to conduct a brief cognitive screen (e.g., MoCA or MMSE). The movement disorders outpatient clinic (Site A) employed a multidisciplinary team, including nurses and allied health that could assist with cognitive screens. Whereas the neurology outpatient clinic (Site B) only had a registrar for such support.

“*The way the clinic is set up, they have a nurse who's an enrolled nurse…and she's also qualified to do MoCA. So the other clinicians, if they're concerned about a patient's cognition, they can just ask her and she'll do a MoCA.”* (Nurse, Staff ID01, quote represents a perceived enabler)

“*I'm lucky enough to have a registrar at [clinic], so sometimes they'll be the ones doing it if they're seeing the patient, but otherwise it would just be me. I don't have any other sort of like nursing staff that would be able to assist with that.”* (Neurologist, Staff ID02, quote represents a perceived enabler and barrier)

Variability across types of healthcare services was also noted. For example, where clinicians worked across both public and private clinics, they were more likely to refer for cognitive assessments in private practice due to the greater availability of neuropsychologists.

“*Access to neuropsychology's quite difficult publicly so there's very long waiting times. It's years since I’ve tried. And except for a case with a really difficult diagnostic dilemma, I don’t intend to do that. In private practice, there's three or four neuropsychologists who I share quite a few patients with and got to know and I just tend to send referrals to one or some of them, depending on their availability.”* (Psychiatrist, Staff ID12, quote represents a perceived barrier and enabler)

In general, it was reported there were insufficient neuropsychologists to conduct in-depth cognitive assessments for people living with PD outside the context of assessment for deep brain stimulation (DBS), and long waitlists were therefore in place.

“*It could be quite a while before they had an in-depth cognitive assessment. Three to six months. That is a different story with our deep brain stimulation patients. They are usually seen fairly quickly, I'd say within one to three months.”* (Nurse, Staff ID08, quote represents a perceived barrier and enabler)

#### Clinician decision-making processes to screen or refer for neuropsychological assessment vary

The decision to conduct a brief cognitive screen with patients living with PD was mostly based on concerns about cognitive changes raised by a clinician, nurse, patient or family caregiver.

“*I might have concerns about their cognition before they come to see us at clinic. I might do all the assessments out there, just so it's all ready and done, and we can get them through a little bit quicker with treatment and planning.”* (Nurse, Staff ID08, quote represents a perceived enabler)

In addition, conducting a MMSE was a requirement for prescribing cholinesterase inhibitors, a drug treatment commonly prescribed for Alzheimer's disease but with efficacy for cognitive decline and hallucinations in Lewy body or Parkinson's disease dementia.

“*But that's actually a practical reason why we might need to do an MMSE in clinic. So it's a long way about why we're doing it. When we bring up to get an authority prescription, we have to provide an MMSE score. So that's the reason sometimes when someone comes in with hallucinations and we're prescribing it. That will do an MMSE. Just for that, if nothing else.”* (Neurologist, Staff ID06, quote represents a perceived enabler)

Decisions to refer for a comprehensive neuropsychological assessment were more variable, however. Outside of assessing eligibility for DBS, referrals were made only for complex cases. These decisions were linked to the lack of neuropsychologists and long waiting times described previously.

“*But of course the reality is, we just have one [neuropsychologist], and it takes her a long time to do a full assessment. So, we very much do that on the more complex end of the market. That is not pointing us in a clear diagnostic direction”.* (Geriatrician, Staff ID07, quote represents a perceived barrier)

Other considerations such as the patient's age (i.e., of working age or not) and stage of cognitive decline (i.e., mild, moderate or severe), were often factored into the decision, as an in-depth neuropsychological assessment may take three hours or more to complete.

“*I tend to reserve more thorough neuropsychological testing for the younger patients, especially those who are working age. I haven't really used it that much in the older patients.”* (Neurologist, Staff ID02, quote represents a perceived enabler and barrier)

#### The impact of undergoing neuropsychological assessments on people living with PD

Many participants discussed how neuropsychological testing could be stressful for the person with PD, and the results confronting when cognitive decline or dementia was identified.

“*It's good to know where you sit on a baseline and how you're progressing. And all that's wonderful until you get to the point where the information says that you're going downhill. How you deal with that, at that point of time of identifying that there is this cognitive decline or indications of cognitive decline.”* (Person living with PD, ID05, quote represents a perceived enabler and barrier)

“*They can get very stressed and anxious doing those assessments too. So, you know they certainly none of them like particularly having the cognitive assessment prior to their DBS surgery because they have to sit there for three hours and it's very detailed. I think you've got to be careful not to cause more stress and anxiety.”* (Nurse, Staff ID01, quote represents a perceived barrier)

Personal choice in being tested and/or receiving results and having family support available, were deemed extremely important. Some participants with PD stated they would prefer not to know their results.

“*I’m a bit wary of that. Because I actually don’t want to know. I’m a bit of an ostrich. I don’t want to know that I’m getting worse. I’d prefer to find out much later on, know that you’re on this scale now, and in two years’ time you’re going to be demented* [sic]*.”* (Person living with PD, ID04, quote represents a perceived barrier)

Conversely, other participants with PD perceived that assessment results validated their experiences. This could help them, and their families understand the cognitive changes they were experiencing.

“*I did a five-hour appointment and reporting process with a neuropsychologist, which I guess it validated some of my experience. It gave me a lot of insight into some of the cognitive changes that I was experiencing.”* (Person living with PD, ID01, quote represents a perceived enabler)

#### Uncertainty over benefits of cognitive assessment in changing clinical management

Clinicians discussed whether there were any benefits of cognitive assessments in terms of how it might change their clinical management and treatment. Some of this discussion related to how assessment results might influence the prescribing or de-prescribing of medication.

“*I think often rapidly accessible cognitive data may be really useful as to part of our assessment of where we go with people. I've talked about adding medicine, sometimes that might be part of our decisions of removing medications”.* (Geriatrician, Staff ID07, quote represents a perceived enabler)

Uncertainty about the value of undergoing a cognitive assessment was discussed by one of the participants living with PD, who wanted more information about the potential benefits and what would happen differently as a result.

“*I think it would be more compelling, both for neurologists, for patients, and for carers, if you could give some specific examples of the positive consequences that could from a cognitive assessment. Not just for monitoring, or research, but show me as a patient, or show me as a carer, what would happen differently as a result; give some case examples. I’m much better informed about cognitive assessment than most members of the public, but I don’t know what I could get out of it.”* (Person living with PD, ID22, quote represents a perceived enabler and barrier)

For other participants living with PD, perceived benefits were discussed in terms of being more proactive about accessing potential treatments and support to manage any cognitive decline at an earlier stage. It should be noted that current PD medications are largely symptomatic.

“*Some of the medications that are available will halt the progression, hopefully. So, you’re better off getting onto them earlier than later”.* (Person living with PD, ID04, quote represents a perceived enabler)

“*If people don’t know what is happening to me, well, then how can they either suggest things or take it into account, or, you know, all of those things that could have a bearing on quality of life.”* (Person living with PD, ID08, quote represents a perceived barrier and enabler)

#### Perceived advantages of PDCogniCare over current practice

The proposed ‘PDCogniCare’ platform was discussed in terms of its advantages or disadvantages over current clinical practice. Clinicians and neuropsychologists indicated they would not be motivated to use it unless there were demonstrable benefits. For example, this could be facilitating more timely access to assessment results and treatments.

“*Having results that are easily accessible and come back in a timely fashion. And also listing recommendations for treatment as well from the psychologist. So those probably the things that I'd be interested in.”* (Neurologist, Staff ID02, quote represents a perceived enabler)

“*I think one thing that could be useful actually is having a metric like MMSE scores and these things which the PBS kind of require, that can be built into what the neuropsychologists do, that could save time for neurologists who don’t tend to do them, so it might actually facilitate access to the PBS listed medications that we might tend to use.”* (Psychiatrist, Staff ID12, quote represents a perceived enabler)

A further potential benefit would be the avoidance of duplicating cognitive testing across different settings, such as in-patient wards and out-patient clinics. That would require access to the platform by multiple health professionals as well as settings, however.

“*Having that central point of storing results so people could access them in a uniform and presumably quick and easy way would be super helpful and may even, stop duplication of tests, you know. So oh, somebody else did it a week ago or a month ago, a neuropsychologist in a hospital did it, then somebody in the community might not have to repeat it.”* (Speech Pathologist, Staff ID16, quote represents a perceived enabler)

Most participants believed that the person with PD and their family members should have access to their cognitive assessment results, as this would promote person-centred care, information sharing and awareness of potential care pathways.

“*When sharing information, and particularly sharing with the patient, and the patient being able to then be given the tools, that they know perhaps what questions they need to ask, based on their results. What direction they need to go to, when they’re going back to their neurologist or their GP to ask questions based on those results.”* (Person living with PD, ID04, quote represents a perceived enabler)

However, it was stipulated that the results needed to be discussed with the person's referring clinician and/or neuropsychologist first, and that any information presented to patients and their families be simplified.

“*I would have concerns about people seeing results before they've had a chance to discuss them. Because if they do have questions, if they do get increased anxiety from the results you know, if they misinterpret them.”* (Neuropsychologist, Staff ID14, quote represents a perceived barrier)

There were also concerns about whether the proposed innovation would increase time burden on clinic staff and whether increased referrals for neuropsychological assessments would overburden current services.

“*At the moment we tend to only get referred to more severe cases, so if we’re suddenly being referred lots and lots more people then, whether we have the capacity to suddenly service all of that extra work, I'm not sure.”* (Neuropsychologist, Staff ID11, quote represents a perceived barrier)

Finally, various clinicians thought that ‘PDCogniCare’ might be more useful for outreach services in regional, rural and remote areas. Others suggested greater use in private practice, but only if the platform improved access to cognitive testing at minimal cost to the patient. Primary care was also discussed as a useful setting as most patients with PD see their GP on a regular basis.

### Recommended implementation strategies

Using the CFIR-ERIC Matching Tool, the following were recommended as high priority and highly feasible strategies (i.e., selected by >60% of implementation experts as appropriate for addressing an identified barrier), that could be used to mitigate barriers for the implementation of ‘PDCogniCare’ (see Additional file 4 for further detail).

***Education and training***: Key barriers were clinician uncertainty about the benefits of cognitive assessment in PD in general and the proposed innovation more specifically, as well as lack of clinician knowledge and training regarding best practice guidelines. Strategies to address these barriers included: the i) development of ‘PDCogniCare’ educational materials (e.g., Best Practice Guidelines for Cognitive Assessment in PD, Neuropsychological Assessment Toolkit for PD, and improved consumer and clinician information); ii) conduct of educational meetings for each stakeholder group (movement disorder and neurology clinic staff, neuropsychologists); and iii) conduct of ongoing training (repeat training for new staff and those on clinical rotation).

***Additional resource allocation***: Lack of clinic resources in terms of time and qualified/trained staff to conduct brief and in-depth cognitive assessments was perceived to be a major barrier. The recommended strategy was to access new funding and allocation of resources. This could be achieved by applying for additional grants to fund dedicated staff time (e.g., neuropsychologists for PD assessments) and utilising post-graduate neuropsychology interns and externs as part of their required clinical placements. The use of telehealth clinics for remote assessments may also address resource constraints in more regional areas.

***Support and feedback:*** Strategies to promote readiness for and use of ‘PDCogniCare’ included: i) identifying and preparing champions at each clinic (movement disorders, neurology, psychology) to support and drive implementation; ii) organizing clinician implementation team meetings at each site to provide feedback and resolve any issues; iii) providing local technical assistance with IT and workflow support, and maintaining system security for the ‘PDCogniCare’ platform; and iv) audit and feedback mechanisms to monitor and evaluate usage of the ‘PDCogniCare’ platform.

***Patient/consumer advocates:*** The active involvement of patients and consumers was identified as an enabler to the use of ‘PDCogniCare’ and included: i) preparing patients/consumers to be active participants in their care (e.g., engaging with local PD support groups); and ii) obtaining and using patients/consumers and family feedback on ‘PDCogniCare’ (e.g., developing opportunities for feedback regarding any significant benefits or issues experienced, and communicating these findings to clinicians, other patient/consumers and their families).

## Discussion

This qualitative inquiry was conducted to assess the context for implementing an innovation called ‘PDCogniCare’. ‘PDCogniCare’ aims to provide guidance on best practice for cognitive assessments in PD, manage referrals and information sharing, and track cognitive assessments for early and improved diagnosis of dementia. Our findings provide insights into neurology, movement disorder and neuropsychology clinic practices, as well as barriers and enablers for delivering and receiving cognitive assessments in PD more broadly.

Several barriers within the TDF constructs of *knowledge*, *skills* and *professional roles* (c/f CFIR domains *individual characteristics; knowledge and beliefs*) were identified. Limited discussion about cognitive impairment between people living with PD, family caregivers and clinicians, was identified as a key barrier to understanding cognitive issues and how to manage or prepare for any future decline. Further, it limited access to neuropsychological assessments and subsequent psychological treatment and care. Inadequate information about cognitive changes and lack of psychological support has also been reported by people living with PD in the UK.^3,6^ As a result, people living with PD and their families are having to seek out information and services themselves, either online, via research studies or by self-referral to private practitioners.^6^ Inequity of access to cognitive assessment, treatment and care may therefore be exacerbated and limited to those with the necessary knowledge, skills and financial resources.^34^

The underlying reasons for this lack of information and discussion were multifaceted, and perspectives varied. Some participants perceived neurology and movement disorders clinicians as not engaged in the cognitive side of PD (*individual characteristics; knowledge and beliefs*). Other studies have reported that PD-related cognitive impairment is poorly understood by health care professionals, with perceived stigma around dementia making such conversations difficult.^6^ However, the clinicians in our study reported several other reasons, including lack of time in a 20-minute annual follow-up consultation (*resources*), not wanting to cause anxiety for patients with PD, and not seeing the benefits of a cognitive assessment in changing clinical management (*beliefs about consequences*). Existing Movement Disorders Society guidelines recommend cognitive evaluation (brief or comprehensive) for PD-dementia and PD-MCI diagnosis.^35,36^ Accurate diagnosis of cognitive disorders is critical to access evidence-based pharmacological and non-pharmacological treatment options, as well as post-diagnostic care pathways.^37^

Decision-making to refer for in-depth neuropsychological assessment also varied. Most referrals were made either to confirm eligibility for DBS or for assessing the most complex cases; neuropsychologists reported receiving very few referrals for people living with PD outside of these circumstances. The person's age and stage of cognitive impairment were also considered, with working age people living with PD and those in the earlier stages of decline more likely to be referred (*decision processes*). Other considerations included the requirement to conduct an MMSE for the purposes of accessing pharmacological treatment (*beliefs about consequences*). These variations might partially be accounted for by the mixed awareness of clinical diagnostic guidelines for cognitive impairment and dementia in PD (*knowledge, evidence*).^35,36,38^ However, established guidelines offer limited recommendations regarding the process of neuropsychological evaluation, for example how often assessments should be conducted, or referral pathways for people experiencing cognitive decline.^15^

Key barriers were also related to the resources available within a specific clinical context (*environmental context and resources*). Variations between settings were noted (e.g., public versus private clinics, metropolitan versus regional and rural), in terms of available staff and time for conducting cognitive screens and comprehensive neuropsychological assessments. Some public neurology and movement disorders clinics were able to offer MoCA or MMSE cognitive screens where trained PD nurses or registrars were available. Even then, resource limitations meant that cognitive assessments were not offered routinely to everyone to determine a baseline; rather only where specific concerns had been raised. It was noted that there are insufficient neuropsychologists available within the public health system to deliver in-depth comprehensive assessments and long waiting times can be experienced. The private healthcare system may offer greater access to neuropsychologist assessments; however, this is limited to those who can afford to pay. The identified barriers relating to clinician and health care professional knowledge and resources to deliver cognitive assessments in PD are likely to be worse in low to middle income countries, where there is a greater shortage of trained professionals, inadequate infrastructure and limited funding available.^39^

Certain enabling factors were identified, which may mitigate some of these barriers (*relative advantage, beliefs about consequences*). Participants perceived that the proposed ‘PDCogniCare’ innovation may facilitate greater access to cognitive assessments, post-diagnostic treatments and support options via referral management, education and information within one central and integrated system.^21^ Recommended implementation strategies included embedding newly developed best practice guidelines alongside education and training for clinical and allied health staff, which may help reduce variation in the evaluation, diagnosis and management of cognitive impairment and dementia in PD.^14^ Clear information about cognitive impairment, as well as the implications and benefits of any assessments, could be downloaded for people living with PD and their families to assist with forward planning and informed decision-making.^14^ This information was identified as vital, due to the potential anxiety of undergoing cognitive testing and receiving a diagnosis of PD-MCI or PD dementia (*emotion*). There was widespread agreement that people living with PD should only be provided access to their test results following discussion with a clinician, due to the impacts associated with diagnosis. Identified benefits of sharing access with other health professionals (e.g., GP, speech pathologist, physiotherapist, occupational therapist), included avoiding duplication of cognitive tests across healthcare settings and promoting more patient-centred, integrated and holistic care planning. Key to implementation will be demonstrating these benefits to clinicians, allied health professionals, people living with PD and their families via audit and feedback mechanisms. Involving patients/consumers and their families in providing feedback to clinicians may also address concerns about benefits of cognitive assessments (*beliefs about consequences*). This will be examined via a future implementation-effectiveness trial, with inbuilt cost-consequence analysis.

Staff resource issues are more difficult to mitigate, however, it was thought that providing workflow support, streamlined referral management, summarised report presentation and visualization may reduce time burdens for clinicians and health professionals (*innovation; design*).^21^ A key barrier was the lack of trained neuropsychologists to conduct in-depth assessments for people living with PD following referral (*readiness for implementation; available resources*).^40^ Securing additional resources (e.g., via grant funding) was identified as a mitigating strategy here. In addition, ‘PDCogniCare’ proposes utilising a research and training integrated clinical model that can build capacity by linking PD clinical services with postgraduate neuropsychology trainees to deliver cognitive screens and assessments.^40^ This approach has been successfully applied in the US, with clinical mental health counselling trainees completing internships within PD clinics.^40^ Further, incorporating options for telehealth delivery of cognitive assessments may improve access and reduce costs for those living in regional and rural areas, or unable to access clinics in-person due to mobility and other health issues.^21^ The potential combined benefits of ‘PDCogniCare’ may thus reduce inequities in healthcare access for people living with PD and their families.^34^

### Strengths and limitations

A major strength of this study is the range of perspectives, roles and experiences obtained, including various clinicians, allied health professionals and people living with PD. Our purposive sampling strategy meant that we obtained more clinician and healthcare professionals’ perspectives than those of people living with PD, which may have impacted the themes constructed. Whilst we did not aim to include caregivers in this study, one joint interview was held with a person living with PD and their partner. We acknowledge the lack of other caregiver perspectives as a limitation in the study. However, we have completed additional work focusing solely on the perspectives of people living with PD and their caregivers in a separate but related study reported elsewhere.^41^ We also acknowledge that participants were recruited from neurology, movement disorders, psychology and psychiatry clinics within two metropolitan health services in Australia only. Therefore, findings may not be generalizable to rural and remote or other healthcare settings.

Finally, as a pre-implementation study, we acknowledge that the findings are reflective of participants’ perceptions and anticipated impacts of ‘PDCogniCare’ rather than observed changes in practice.

## Conclusion

This study provides insights into barriers and enablers for the delivery of cognitive assessments for people living with PD, within the context of implementing an innovation ‘PDCogniCare’. The study was informed by two widely utilised implementation science frameworks (CFIR and TDF), to more fully understand behaviour change within healthcare organisations. We used the CFIR-ERIC Matching Tool to develop key implementation strategies to mitigate identified barriers and leverage enablers. We will subsequently apply these strategies in a future implementation-effectiveness trial of ‘PDCogniCare’.

## Supplemental Material

sj-docx-1-pkn-10.1177_1877718X261440707 - Supplemental material for Barriers and enablers to cognitive assessment in Parkinson's disease: A qualitative contextual inquiry Supplemental material, sj-docx-1-pkn-10.1177_1877718X261440707 for Barriers and enablers to cognitive assessment in Parkinson's disease: A qualitative contextual inquiry by Deborah Brooks, Kirstine Shrubsole, Deepa Sriram, Jihyun Yang, Leander K. Mitchell, Peter Worthy, Elton Lobo, Dana Pourzinal and Nadeeka N. Dissanayaka in Journal of Parkinson's Disease

sj-docx-2-pkn-10.1177_1877718X261440707 - Supplemental material for Barriers and enablers to cognitive assessment in Parkinson's disease: A qualitative contextual inquiry Supplemental material, sj-docx-2-pkn-10.1177_1877718X261440707 for Barriers and enablers to cognitive assessment in Parkinson's disease: A qualitative contextual inquiry by Deborah Brooks, Kirstine Shrubsole, Deepa Sriram, Jihyun Yang, Leander K. Mitchell, Peter Worthy, Elton Lobo, Dana Pourzinal and Nadeeka N. Dissanayaka in Journal of Parkinson's Disease

sj-docx-3-pkn-10.1177_1877718X261440707 - Supplemental material for Barriers and enablers to cognitive assessment in Parkinson's disease: A qualitative contextual inquiry Supplemental material, sj-docx-3-pkn-10.1177_1877718X261440707 for Barriers and enablers to cognitive assessment in Parkinson's disease: A qualitative contextual inquiry by Deborah Brooks, Kirstine Shrubsole, Deepa Sriram, Jihyun Yang, Leander K. Mitchell, Peter Worthy, Elton Lobo, Dana Pourzinal and Nadeeka N. Dissanayaka in Journal of Parkinson's Disease

sj-docx-4-pkn-10.1177_1877718X261440707 - Supplemental material for Barriers and enablers to cognitive assessment in Parkinson's disease: A qualitative contextual inquiry Supplemental material, sj-docx-4-pkn-10.1177_1877718X261440707 for Barriers and enablers to cognitive assessment in Parkinson's disease: A qualitative contextual inquiry by Deborah Brooks, Kirstine Shrubsole, Deepa Sriram, Jihyun Yang, Leander K. Mitchell, Peter Worthy, Elton Lobo, Dana Pourzinal and Nadeeka N. Dissanayaka in Journal of Parkinson's Disease
