# Supplementary material for: Barriers and enablers to cognitive assessment in Parkinson's disease: A qualitative contextual inquiry
Source: J Parkinsons Dis. 2026 Apr 27:1877718X261440707. Online ahead of print. doi: 10.1177/1877718X261440707 (PMC13435214; doi:10.1177/1877718X261440707)
Supplement: Supplementary file 2 [file sj-docx-2-pkn-10.1177_1877718X261440707.docx]

**Interview Topic Guide**

**(Interviews with Lived Experience Experts)**

**Enhancing Utility of Neuropsychological Evaluation for Earlier and Effective Diagnosis of Dementia in Parkinson’s disease (PDCogniCare)**

**Research aims:**

- To understand usual care and processes with regard to cognitive assessment and the use of assessment results within clinical services (e.g., neurology, Movement Disorders Clinics, psychology services, geriatric and memory clinic services etc)
- To identify the needs of people with Lived Experience of Parkinson’s disease and their care partners, with regard to cognitive assessment
- To identify perceptions of factors (i.e., potential barriers and enablers) that may impact the implementation of a new system to refer people with Parkinson’s disease for cognitive assessments by The University of Queensland post-graduate psychologists (PDCogniCare).

**Briefing/Introduction**

- Researcher to introduce self and background
- Thank participant for taking part in research (e.g., “***Thank you for your involvement in this research study. We would like to understand your experiences of attending Parkinson’s / Neurology / Movement Disorders clinics and of cognitive assessments.*** ***A cognitive assessment is a test of your brain's abilities, such as thinking, memory, language, and judgment.”***
- Explain the purpose of the research:
- I would like to talk to you about:

(i)	Your experiences of attending specialised clinics other than your GP to discuss and monitor your Parkinson’s disease (e.g., Parkinson’s or Neurology or Movement Disorders clinics), either in-person or via telehealth;

(ii)	The time and resources you need for attending clinics in person or via telehealth;

(iii)	Your experiences of, and beliefs about, cognitive assessments in Parkinson’s disease;

(iv)	Your views about a new system to help clinicians to track cognitive assessments.

(v)	How you feel about early diagnosis of cognitive impairment or dementia in Parkinson’s disease.

- Explain interview process:
- The interview will probably go for 1 hour depending on how much discussion arises.
- If at any point you would like to take a break, just let me know.
- The interview will be recorded with your consent.
- You do not have to answer every question and can cease the interview at any time if you wish.
- The researcher will take some notes during the interview to keep track of the main points
- Do you have any questions for me at this stage?

**Demographic information**

Age______________________________________________________________________

Gender___________________________________________________________________

Person living with PD	Y / N

Family care partner Y / N

If yes, relationship to person with PD___________________________________________

Year of diagnosis of PD_____________________________________________________

Do you have any other diagnosis other than PD? _________________________________

If so, please list:

**Research topics**

**Experience of attending PD clinics and assessments**

How often do you attend PD clinics?

Do you always attend in-person or have you had any telehealth appointments?

How do you find attending the PD clinics? (e.g., helpful, tiring etc) *TDF domain Emotion*

What resources do you need to attend the clinics (e.g., transport, care partner, time off work etc?) *TDF domain ENV – context and resources*

Who do you normally see at the clinics (e.g., MD nurse, clinician, allied health?)

What types of assessment do you tend to have at the clinics?

Who else is involved in your PD care?

**Experience of and attitudes towards cognitive assessments in PD**

Have you had any cognitive assessments conducted at / or referred by the PD clinic?

If yes, who was the assessment conducted by? Was it in-person or via telehealth? (please give as much detail as possible)

If yes - What was your experience of having a cognitive assessment (what did you do, how did you feel?) *TDF domain Emotions*

If yes, how long did you wait for the results? Who talked to you about the results? What (if anything) happened next?

If no – What do you think / how do you feel, about having a cognitive assessment in the future? (in-person and / or via telehealth) *TDF domain motivations*

Do you think cognitive assessments should be routinely offered to people living with Parkinson’s disease? (why or why not - *Keep this quite open and try to just listen here- listen for different domains that may come up spontaneously)* *TDF domain Motivations*

If yes, who do you think should be conducting cognitive assessments? (e.g., neurologist, psychologist, nurse, other)

If yes, how often should people with PD be offered a cognitive assessment?

How do you feel about cognitive assessments conducted via telehealth (e.g., teleconferencing such as zoom rather than in-person?) *TDF domain opportunity, capability, knowledge, physical*

**Views about the new PDCogniCare system to help clinicians track cognitive assessments.**

**Play the video that shows what PDCogniCare is.**

What are your initial thoughts about the PDCogniCare ‘platform’? *Keep this quite open and try to just listen here- listen for different domains that may come up spontaneously)*

- What concerns do you have about this idea?
- What information would you like to be able to see?
- What do you think is good about this idea?
- What could make it better?
- Would you like to have access to any cognitive assessments that were conducted for you / or your care partner? (why or why not? What would you use it for? Would you share it with others? Who?) *TDF domains: incentives, motivations, consequences, control / empowerment*

**Beliefs and attitudes about early diagnosis of mild cognitive impairment or dementia in Parkinson’s disease**

- How do you feel about the early diagnosis of mild cognitive impairment or dementia in Parkinson’s disease? (e.g., is it important or beneficial, why or why not?) *TDF domains: emotions, beliefs, motivations etc*

What are the potential implications of a diagnosis of mild cognitive impairment or dementia in PD? (e.g., Issues surrounding diagnosis and post-diagnostic support) *Keep this quite open and try to just listen here- listen for different domains that may come up spontaneously) TDF domain: beliefs about consequences*

**De-briefing**

**Aims:** To conclude the interview; to allow a forum for any questions

- Is there anything else about that we haven’t discussed that you think is important?

***Direction to support services as appropriate:***

- Dementia Australia National Dementia Helpline: 1800 100 500
- Beyond Blue Helpline: 1300 22 4636
- Lifeline crisis support: 13 11 14
