# Supplementary material for: Barriers and enablers to cognitive assessment in Parkinson's disease: A qualitative contextual inquiry
Source: J Parkinsons Dis. 2026 Apr 27:1877718X261440707. Online ahead of print. doi: 10.1177/1877718X261440707 (PMC13435214; doi:10.1177/1877718X261440707)
Supplement: Supplementary file 3 [file sj-docx-3-pkn-10.1177_1877718X261440707.docx]

**Interview Topic Guide**

**(Interviews with MDC Health professionals**

**Enhancing Utility of Neuropsychological Evaluation for Earlier and Effective Diagnosis of Dementia in Parkinson’s disease (PDCogniCare)**

**Research aims:**

- To understand usual practice and processes in regards to cognitive assessment and use of assessment results within Movement Disorders Clinics (MDC)
- To identify stakeholder needs in regards to cognitive assessment in PD
- To identify perceptions of what factors (i.e., potential barriers and enablers) would impact implementation of PDCogniCare.

**Briefing/Introduction**

- Thank participant for taking part in research (e.g., “***Thank you for your involvement. We would like to get your input into the current practices here and understand what the needs would be for implementing a platform to help refer for and track cognitive assessments in PD (PDCogniCare platform….”)***
- Researcher to introduce self and background
- Explain the purpose of the research:
- I would like to talk to you about your current practice and role within the MDC and how cognitive assessments are conducted and used for people with PD
- I am particularly interested in your perspectives about what the possible barriers and facilitators would be to implementing a new platform called PDCogniCare once it is developed
- You do not have to answer every question and can cease the interview at any time if you wish.
- Explain interview process:
- Interview will probably go for 1 hour depending on how much discussion arises
- If at any point you would like to take a break, just let me know
- Interview will be recorded
- Researcher will take some notes during the interview to keep track of the main points
- Explain importance of wanting to understand the experiences and perspectives of clinicians working in the MDCs 🡪 deidentified 🡪 Be really helpful to have all of your feedback – even if some opinions may not be positive – so that the project team can work to improve the program and also have the best chance of being successfully implemented.
  - Do you have any questions for me at this stage?

**Demographic information & Role in MDC (5 mins)**

Age_________________________________________________________________________________________________

Gender_____________________________________________________________________________________________

Years of clinical practice__________________________________________________________________________

Current role/position & which hospital & service______________________________________________

Grade/Level________________________________________________________________________________________

Years in current role_______________________________________________________________________________

- Tell me about your role in the clinic? *Prompt to go through role in detail*.

**Research topics**

**Usual practice in cognitive assessment within Movement Disorder Clinic**

**(10 mins)**

- How familiar are you with the evidence and recommendations for conducting assessment of cognition in PD? *TDF domains: knowledge*

Tell me about how cognitive assessments for MDC patients are usually conducted in your clinic? *Prompt to go through process in detail*. *CFIR Inner setting current practice*

(Prompts: Who conducts assessments? When are assessments conducted – is there routine timing? What assessments are conducted? How are results used?) *CFIR Inner Setting, Implementation climate, compatibility*

Who do you see has the role of conducting cognitive assessments? *CFIR Inner Setting compatibility*

Can you tell me about the referral processes for cognitive assessments? (how do you refer? How long does it take to get results? Any challenges with getting information/reports back or referring) (*Keep this quite open and try to just listen here- listen for different domains that may come up spontaneously), CFIR Inner setting, implementation climate, compatibility, tension for change*

***Transition to next topic: Now I’m interested in hearing about what you think is needed for the PDCogniCare platform and what it would take to be implemented into this MDC.***

**Needs in regards to routine cognitive assessment in PD and how PDCogniCare may ‘look’ (15 mins)**

- What are your main needs for cognitive assessment for PD? *CFIR Inner setting, tension for change*

**Show the video explaining PDCogniCare**

- What are your initial thoughts about the concept for PDCogniCare? *CFIR intervention characteristics, relative advantage;* I*nner setting, compatibility; Characteristics of individuals, beliefs about intervention etc*
- If you were to use PDCogniCare, what would you need it to do? *CFIR intervention characteristics, adaptability; inner setting, compatibility; Characteristics of individuals, beliefs about intervention etc*
- What training do you need to be able to use the PDCogniCare platform? *TDF Skills*
- What else would you need to be able to use PDCogniCare? *[Probe here into questions about integrating into the software you use already, who would have to approve the use of PDCogniCare?] CFIR characteristics of individuals, self-efficacy; Inner setting, compatibility, available resources etc*

**Potential of implementing/using PDCogniCare in your service (*30 mins)***

***Transition to next topic: Now I’m interested in hearing about what factors would influence whether or not your service would be able to use the PDCogniCare platform***

- Describe the factors that may influence whether or not (name the service) would use the PDCogniCare platform?
- Would using PDCogniCare fit within your role? *TDF professional role*
- What, if any, difficulties would you have in using PDCogniCare platform? (consider patient and clinician burden) *TDF beliefs about capabilities/confidence*
- What do you think would be the benefits of PDCogniCare? (prompt for who? Is it everyone or specific clients)
- Do you think there would be any negative consequences if you weren’t able to use PDCogniCare? *TDF* *Beliefs about consequences*
- Are there any incentives/disincentives for using PDCogniCare? *TDF* *reinforcement*
- How important do you feel it is to routinely use PDCogniCare? *TDF* *intentions*
- How motivated are you to use PDCogniCare with your patients/within your service? *TDF* *Goals*
- How optimistic are you that the patient’s outcomes would be better if you use PDCogniCare? *TDF* *Optimism*
- Is there are time when you would decide not to use PDCogniCare (or do you make a deliberate decision to NOT assess cognition under certain circumstances)? Would you ever forget to use it? *TDF* *memory or decision-making-*
- To what extent would resources (physical resources, human resources, time etc) influence whether you would use PDCogniCare? What other competing demands would be at play? (funding, logistics, access) Technical barriers? *TDF* *Environmental context and resources*
- In what way does the client/family influence your decision to assess cognition or use the results of cognitive assessments? *TDF Social influences*
- How comfortable would you be using PDCogniCare? Are there any aspects of the platform that could create a difficult experience to deal with? *TDF Emotion*
- Would a system for checking whether or not you used PDCogniCare be helpful? Would a protocol facilitate the use of PDCogniCare? *TDF Behavioural regulation*

**De-briefing**

**Aims:** To conclude the interview; to allow a forum for any questions

- Is there anything else about that we haven’t discussed that you think is important?
