# Supplementary material for: Barriers and enablers to cognitive assessment in Parkinson's disease: A qualitative contextual inquiry
Source: J Parkinsons Dis. 2026 Apr 27:1877718X261440707. Online ahead of print. doi: 10.1177/1877718X261440707 (PMC13435214; doi:10.1177/1877718X261440707)
Supplement: Supplementary file 4 [file sj-docx-4-pkn-10.1177_1877718X261440707.docx]

**Additional File 4. Identified barriers mapped to ERIC strategies and CFIR domains**

| **Identified Barriers** | **ERIC Strategies to Mitigate Barriers** | | **CFIR Domains** | | | | | | | | | |
| --- | --- | --- | --- | --- | --- | --- | --- | --- | --- | --- | --- | --- |
|  |  |  | **Intervention characteristics** | | | **Characteristics of individuals** | | **Inner Setting** | | | **Outer setting** | **Process** |
|  |  |  | *Evidence strength & quality* | *Cost* | *Relative advantage* | *Knowledge & beliefs* | *Self-efficacy* | *Access to knowledge & information* | *Available resources* | *Goals & feedback* | *Patient needs & resources* | *Reflecting & evaluating* |
| Uncertainty over benefits of cognitive assessment | Education & training | Develop educational materials | X |  |  |  |  | X |  |  |  |  |
| Clinicians not on board with cognitive aspects of PD |  | Conduct educational meetings | X |  | X | X |  | X |  |  |  |  |
| Variability in decision-making to screen or refer for cognitive assessment |  | Conduct ongoing training |  |  |  |  | X | X |  |  |  |  |
| Availability of clinic resources to conduct cognitive screens and assessments | Additional resource allocation | Access new funding |  | X |  |  |  |  | X |  |  |  |
|  |  | Allocation of resources |  |  |  |  |  |  | X |  |  |  |
| Perceived disadvantages of ‘PDCogniCare’ | Support & feedback | Identify & prepare champions | X |  | X | X | X |  |  |  |  |  |
|  |  | Organise clinician implementation team meetings |  |  |  |  |  |  |  | X |  | X |
|  |  | Provide local technical assistance |  |  |  |  | X | X |  |  |  |  |
|  |  | Audit & provide feedback |  |  |  |  | X |  |  | X |  | X |
| Lack of discussion limits access to cognitive assessments | Patient Consumer advocates | Prepare patients/consumers to be active participants |  |  |  |  |  |  |  |  | X |  |
| Impact of cognitive assessment on people with PD |  | Involve patients/consumers and family members |  |  |  |  |  |  |  |  | X |  |
| Uncertainty over benefits of cognitive assessments |  | Obtain and use patients/consumers and family feedback |  |  |  |  |  |  |  |  | X | X |
